# Supplementary material for: Stat3 regulates centrosome clustering in cancer cells via Stathmin/PLK1
Source: Nat Commun. 2017 May 5;8:15289. doi: 10.1038/ncomms15289 (PMC5424153; doi:10.1038/ncomms15289)
Supplement: Supplementary Information — Supplementary Figures [file ncomms15289-s1.pdf]

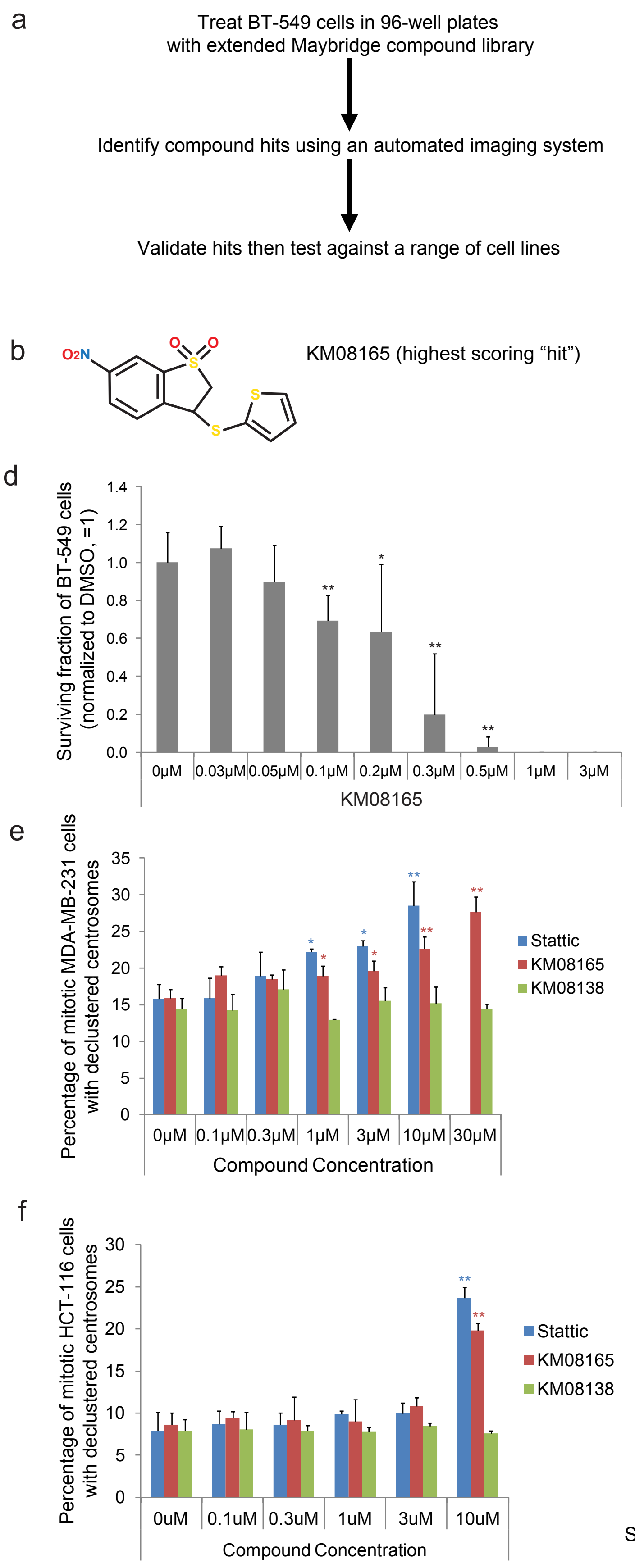

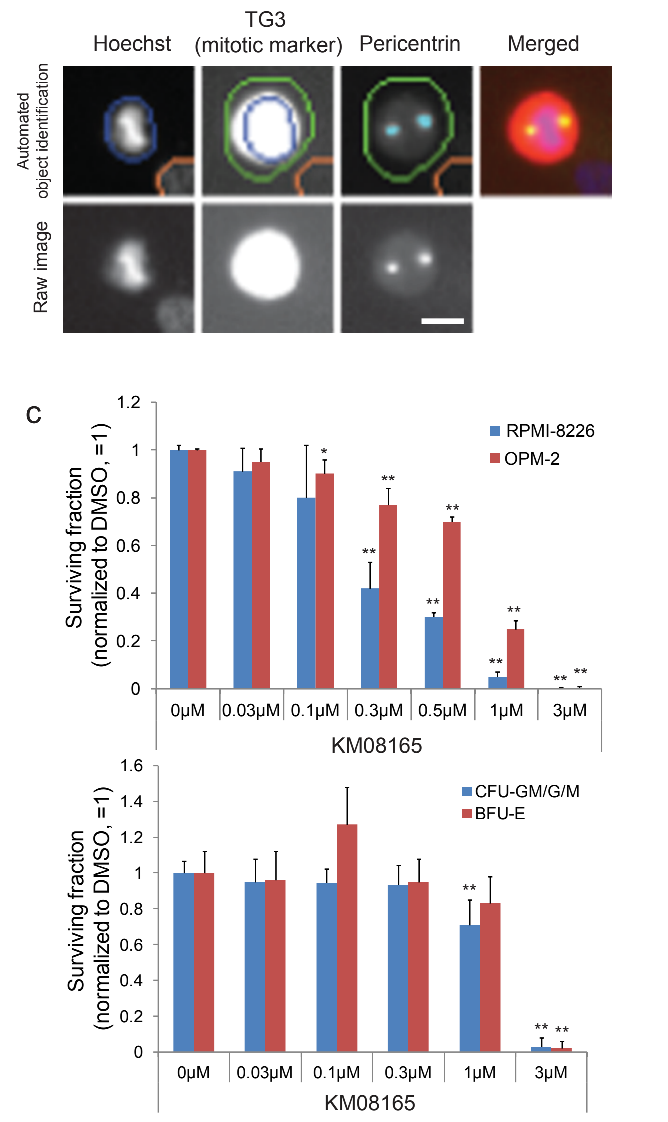

g Metabolic Stability of KM08165 in Human Liver Microsomes

	Time (min)	KM08165 % Remaining	Stattic (µM)
Liver Microsomes with NADPH	0	100 ± 9	BLOQ
	5	83 ± 3	BLOQ
	15	63 ± 3	BLOQ
	30	42 ± 5	BLOQ
	45	30 ± 5	BLOQ (n=2), 2.5 ←
	60	28 ± 5	2.6 ± 0.1 ←
Liver Microsomes without NADPH	60	114	BLOQ
Buffer Alone	0	100 ± 0.3	BLOQ
	60	85 ± 3	BLOQ

BLOQ = below lower limit of quantitation (LLOQ)
LLOQ for Stattic = 2.5 µM

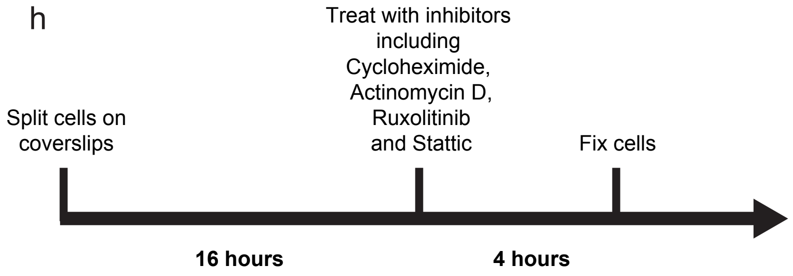

Supplementary Figure 1 Automated screening of centrosome clustering inhibitors. (a) Diagram of the steps performed to screen for new centrosome clustering inhibitors (left panel). Right panel: Automated imaging of BT549 cells. Hoechst was used to locate cells and TG3 staining was used to determine whether the cell was in mitosis. The number of centrosomes was counted using Pericentrin stain. Scale bar, 8 µm. (b) Structure of KM08165, identified in an automated phenotypic screen of centrosome clustering. (c) Colony-forming assay of myeloma cells (top) and normal human primary bone marrow cells (bottom) treated with KM08165. n=3 biological replicates. Statistical significance was tested between untreated and KM08165-treated groups with ANOVA. (d) Clonogenic assay of invasive breast cancer cells (BT-549) treated with KM08165. n=8 biological replicates. Statistical significance was tested between untreated and KM08165-treated groups with ANOVA. (e) Quantification of the percentage of mitotic MDA-MB-231 cells with declustered centrosomes after treatment with Stattic, KM08165 and KM08138. Note that 30µM Stattic treatment was cytotoxic to cells and was not included. n=3 biological replicates, ≥600 cells/data point. Statistical significance was tested between untreated and compound-treated groups with ANOVA. (f) Quantification of the percentage of mitotic HCT-116 cells with declustered centrosomes after treatment with Stattic, KM08165 and KM08138. n=4 biological replicates, ≥800 cells/data point. Statistical significance was tested between untreated and compound-treated groups with ANOVA. (g) UPLC-UV detection of KM08165 degradation and Stattic formation over time using a human liver microsome assay. Conversion to Stattic was detected at quantifiable levels at 45 and 60 minutes (arrows). (h) Schematic timeline of inhibitor treatments. All inhibitory compounds were added to cells for 4 hours and then cells were fixed. *, $p<0.05$; **, $p<0.01$. Error bars represent SEM.

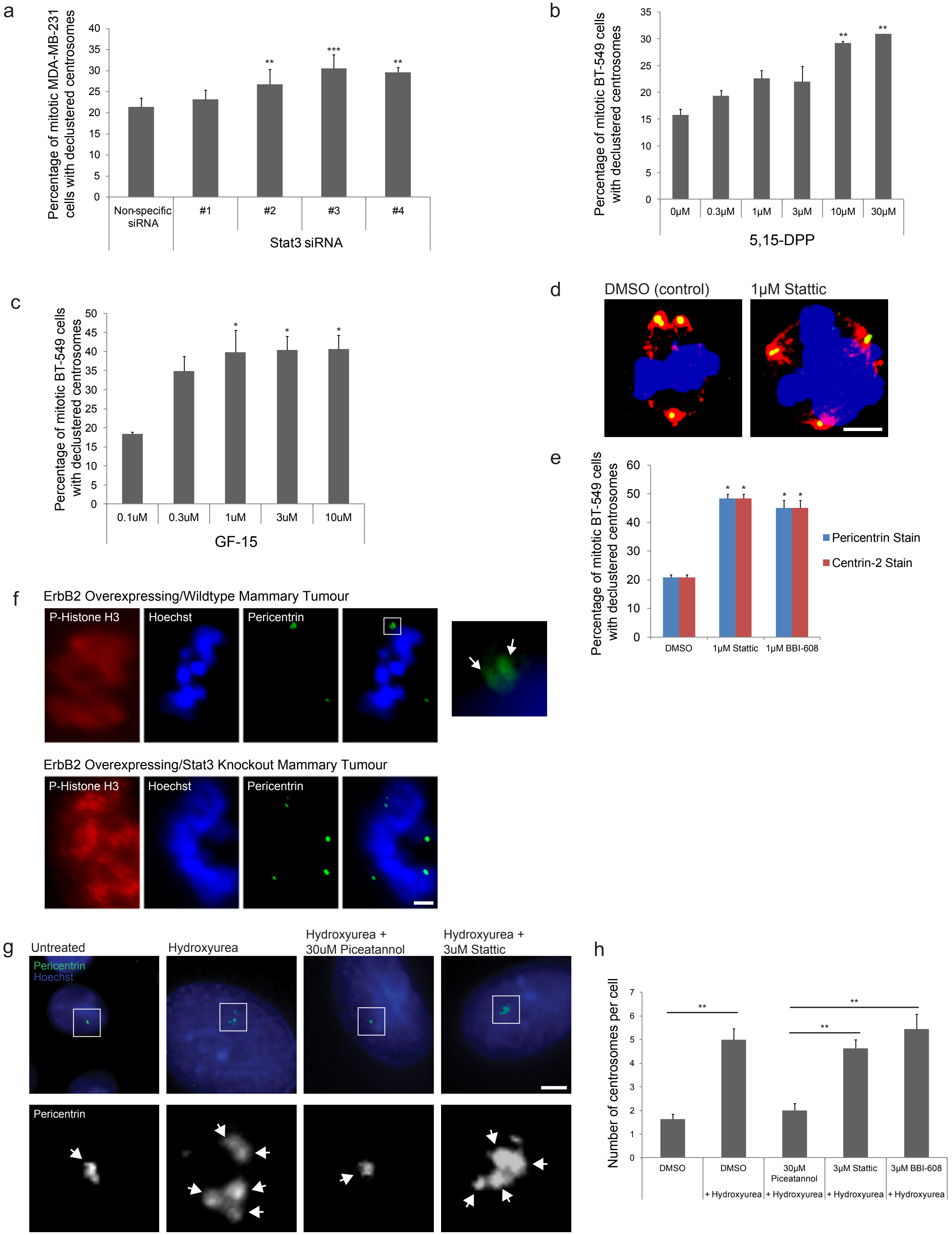
a
Percentage of mitotic MDA-MB-231 cells with declustered centrosomes
40
35
30
25
20
15
10
5
0
**
***
**
Non-specific siRNA
#1
#2
#3
#4
Stat3 siRNA
b
Percentage of mitotic BT-549 cells with declustered centrosomes
35
30
25
20
15
10
5
0
**
**
0μM
0.3μM
1μM
3μM
10μM
30μM
5,15-DPP
c
Percentage of mitotic BT-549 cells with declustered centrosomes
50
45
40
35
30
25
20
15
10
5
0
*
*
*
0.1uM
0.3uM
1uM
3uM
10uM
GF-15
d
DMSO (control)
1μM Stattic
e
Percentage of mitotic BT-549 cells with declustered centrosomes
60
50
40
30
20
10
0
DMSO
1μM Stattic
1μM BBI-608
Pericentrin Stain
Centrin-2 Stain
f
ErbB2 Overexpressing/Wildtype Mammary Tumour
P-Histone H3
Hoechst
Pericentrin
ErbB2 Overexpressing/Stat3 Knockout Mammary Tumour
P-Histone H3
Hoechst
Pericentrin
g
Untreated
Hydroxyurea
Hydroxyurea + 30uM Piceatannol
Hydroxyurea + 3uM Stattic
Pericentrin
Hoechst
Pericentrin
h
Number of centrosomes per cell
7
6
5
4
3
2
1
0
**
**
**
DMSO
DMSO + Hydroxyurea
30μM Piceatannol + Hydroxyurea
3μM Stattic + Hydroxyurea
3μM BBI-608 + Hydroxyurea

Supplementary Figure 2 Further characterisation of Stat3-dependent centrosome clustering. (**a**) Quantification of the percentage of Stat3 siRNA treated mitotic MDA-MB-231 cells with declustered centrosomes. n=4 biological replicates, ≥800 cells/condition. Statistical significance was tested between Non-Specific and Stat3 siRNA-treated groups with ANOVA. (**b**) Quantification of the percentage of 5,15-DPP-treated mitotic BT549 cells with declustered centrosomes. 5,15-DPP is a Stat3 inhibitor. n=3 biological replicates, ≥600 cells/data point. Statistical significance was tested between untreated and 5,15-DPP-treated groups with ANOVA. (**c**) Quantification of the percentage of GF-15-treated mitotic BT549 cells with declustered centrosomes. n=4 biological replicates, ≥800 cells/data point. GF-15 is a previously characterized centrosome clustering inhibitor and was used as a positive control. Statistical significance was tested between untreated and GF-15-treated groups with ANOVA. (**d**) Immunofluorescence images of Pericentrin (red), Centrin-2 (green) and DNA (Hoechst, blue) in mitotic BT549 cells treated with DMSO or Stattic. Scale bar, 5µm. (**e**) Quantification of the percentage of Stattic and BBI-608-treated mitotic BT549 cells with declustered centrosomes using Pericentrin and Centrin-2 to mark centrosomes. n=3 biological replicates, 120 cells/data point. Statistical significance was tested between DMSO (control) and treated groups with ANOVA. (**f**) Immunofluorescence images of Phospho-Histone H3 (mitotic marker, red), DNA (Hoechst, blue) and Pericentrin (green) in mouse tumor sections with Stat3 knocked out. Scale bar, 2µm. Arrows point to 2 clustered centrosomes in the inset. (**g**) Immunofluorescence images of CHO cells treated with Hydroxyurea and Piceatannol or Stattic. Pericentrin (green), DNA (Hoechst, blue). Scale bar, 4 µm. Lower panels: Magnified images of Pericentrin. Centrosomes are highlighted with arrows. (**h**) Quantification of the average number of centrosomes in CHO cells treated with Hydroxyurea and Piceatannol, Stattic or BBI608. n=20 cells quantified, taken from three biological replicates. Statistical significance was tested between the indicated groups with ANOVA. *, $p<0.05$; **, $p<0.01$. Error bars represent SEM.

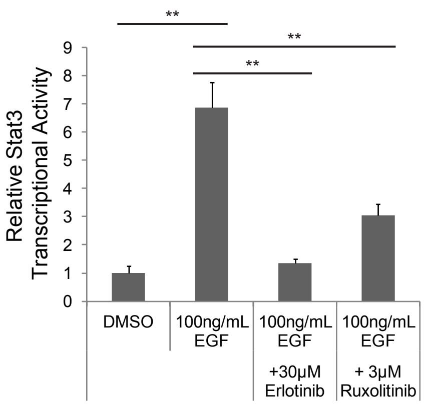

Supplementary Figure 3 Quantification of Stat3 transcriptional activity in BT-549 cells treated with EGF, Erlotinib (EGFR inhibitor) and Ruxolitinib (Jak1/2 inhibitor) using a luciferase reporter assay. Stat3 transcriptional activity was measured relative to a Stat3-independent control reporter. n=4 biological replicates. **, $p<0.01$. Error bars represent SEM. Statistical significance was tested between the indicated groups with ANOVA.

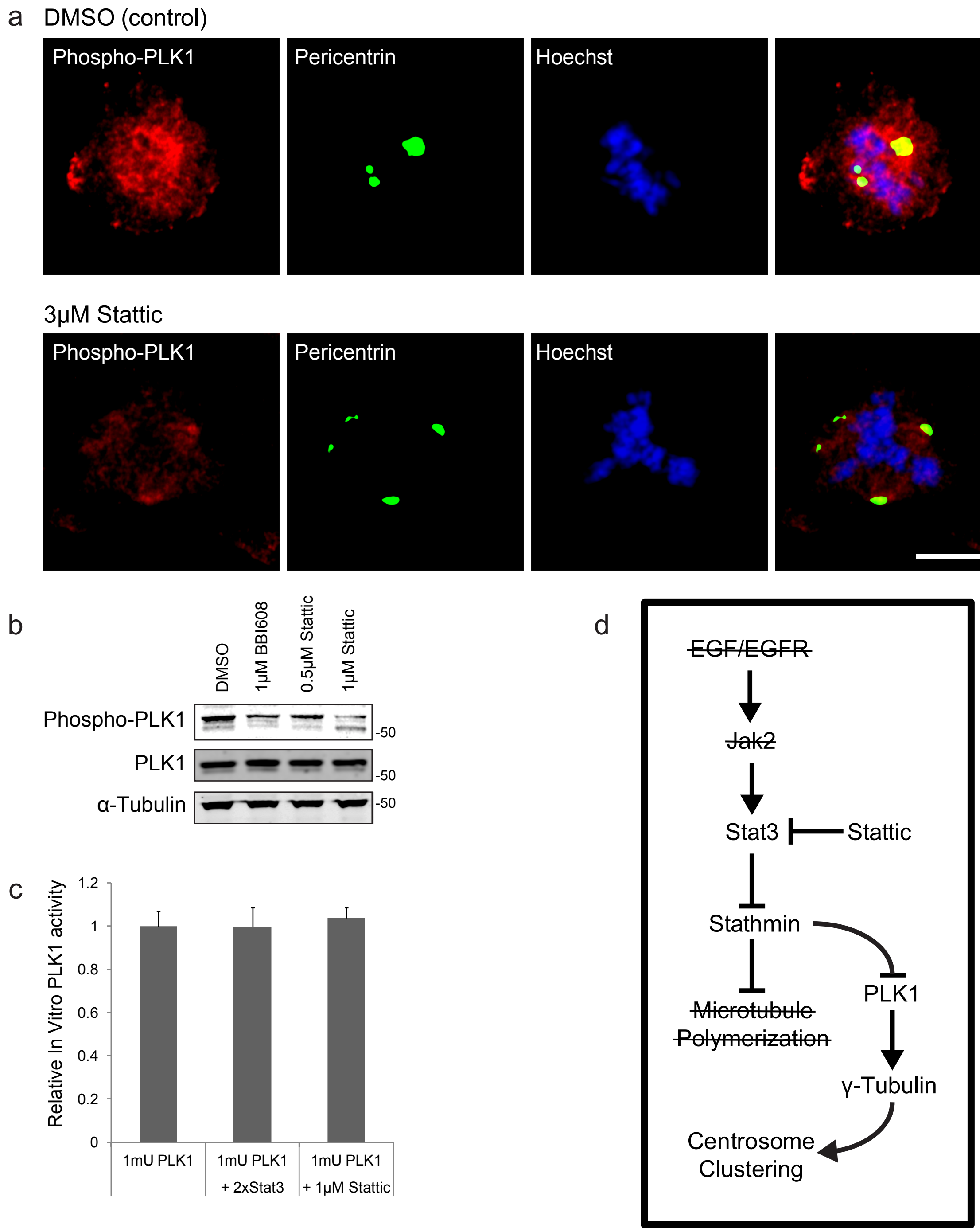

Supplementary Figure 4 Further characterization of Stat3-dependent PLK1 signaling**.** (**a**) Immunofluorescence images of Phospho-PLK1 (red), Pericentrin (green) and DNA (Hoechst, blue) in mitotic BT549 cells treated with DMSO (top) or Stattic (bottom). Scale bar, 8μm. (**b**) Western Blot of Phospho-PLK1 and PLK1 from lysates of MDA-MB-231 cells treated with Stattic or BBI608. Tubulin was used as a loading control. (**c**) Quantification of relative In Vitro PLK1 activity using 1mU PLK1 and either 2xStat3 or 1μM Stattic. n=3 biological replicates. 1mU (milli-Unit)= 1nmole phosphate incorporated min^{-1} mg^{-1}. Error bars represent SEM. (**d**) Diagram of the proposed Stat3-PLK1 centrosome clustering pathway.

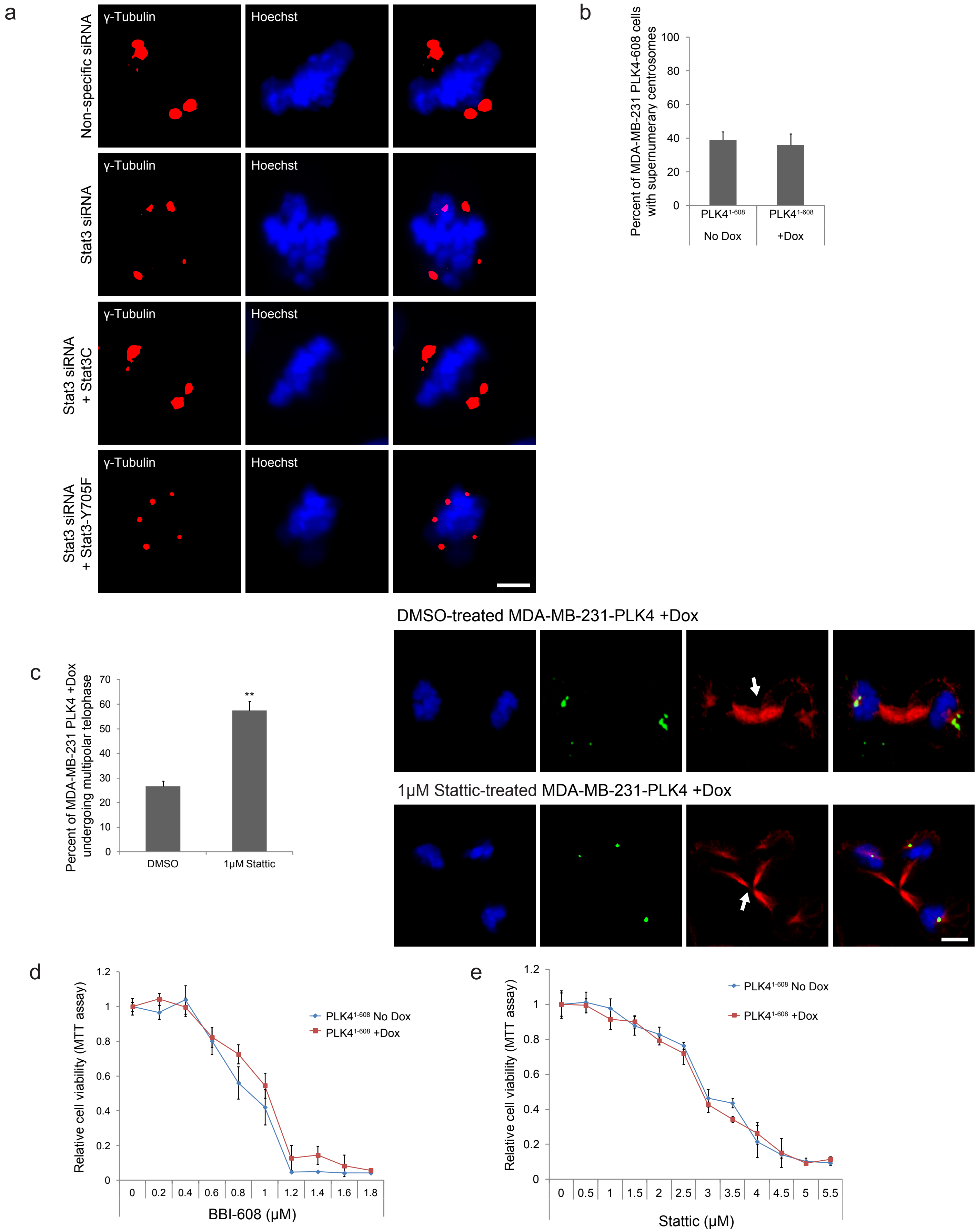

a
Non-specific siRNA
Stat3 siRNA
Stat3 siRNA + Stat3C
Stat3 siRNA + Stat3-Y705F
γ-Tubulin
Hoechst
b
Percent of MDA-MB-231 PLK4-608 cells with supernumerary centrosomes
100
80
60
40
20
0
PLK4 1-608 No Dox
PLK4 1-608 +Dox
c
Percent of MDA-MB-231 PLK4 +Dox undergoing multipolar telophase
70
60
50
40
30
20
10
0
**
DMSO
1μM Stattic
DMSO-treated MDA-MB-231-PLK4 +Dox
1μM Stattic-treated MDA-MB-231-PLK4 +Dox
d
Relative cell viability (MTT assay)
1.2
1
0.8
0.6
0.4
0.2
0
PLK4 1-608 No Dox
PLK4 1-608 +Dox
0
0.2
0.4
0.6
0.8
1
1.2
1.4
1.6
1.8
BBI-608 (μM)
e
Relative cell viability (MTT assay)
1.2
1
0.8
0.6
0.4
0.2
0
PLK4 1-608 No Dox
PLK4 1-608 +Dox
0
0.5
1
1.5
2
2.5
3
3.5
4
4.5
5
5.5
Stattic (μM)

Supplementary Figure 5 γ-Tubulin in Stat3C or Stat3-Y705F cells and cell viability of Stat3 inhibitor treated PLK4-induced cells. (**a**) Immunofluorescence images of γ-Tubulin (red) and DNA (Hoechst, blue) in mitotic BT-549 cells stably expressing Stat3C or Stat3-Y705F and treated with Stat3 siRNA. Scale bar, 4 µm. (**b**) Quantification of the number of doxycycline-induced (Dox) MDA-MB-231-PLK4$^{1\text{-}608}$ cells with supernumerary centrosomes. n=5 biological replicates, 100 cells/condition. (**c**) Left: Quantification of the number of Stattic-treated doxycycline-induced (Dox) MDA-MB-231-PLK4 cells with multipolar telophase relative to the total number of telophase cells. n=4 biological replicates, 120 cells/condition. Right: Representative immunofluorescence images of telophase DMSO-treated and Stattic-treated MDA-MB-231 cells with doxycycline-induced, PLK4-dependent centrosome amplification. Tubulin (red), Pericentrin (green), DNA (Hoechst, blue). Arrows point to cytokinetic bridges (indicative of telophase). Scale bar, 10µm. (**d**) Quantification of cell viability (MTT assay) in BBI-608-treated MDA-MB-231-PLK4$^{1\text{-}608}$ cells with (PLK4$^{1\text{-}608}$+Dox) or without (PLK4$^{1\text{-}608}$ No Dox) doxycycline-induced PLK4$^{1\text{-}608}$ expression. n=6 biological replicates. (**e**) Quantification of cell viability (MTT assay) in Stattic-treated MDA-MB-231-PLK4$^{1\text{-}608}$ cells with (PLK4$^{1\text{-}608}$+Dox) or without (PLK4$^{1\text{-}608}$ No Dox) doxycycline-induced PLK4-608 expression. n=6 biological replicates. Error bars represent SEM.

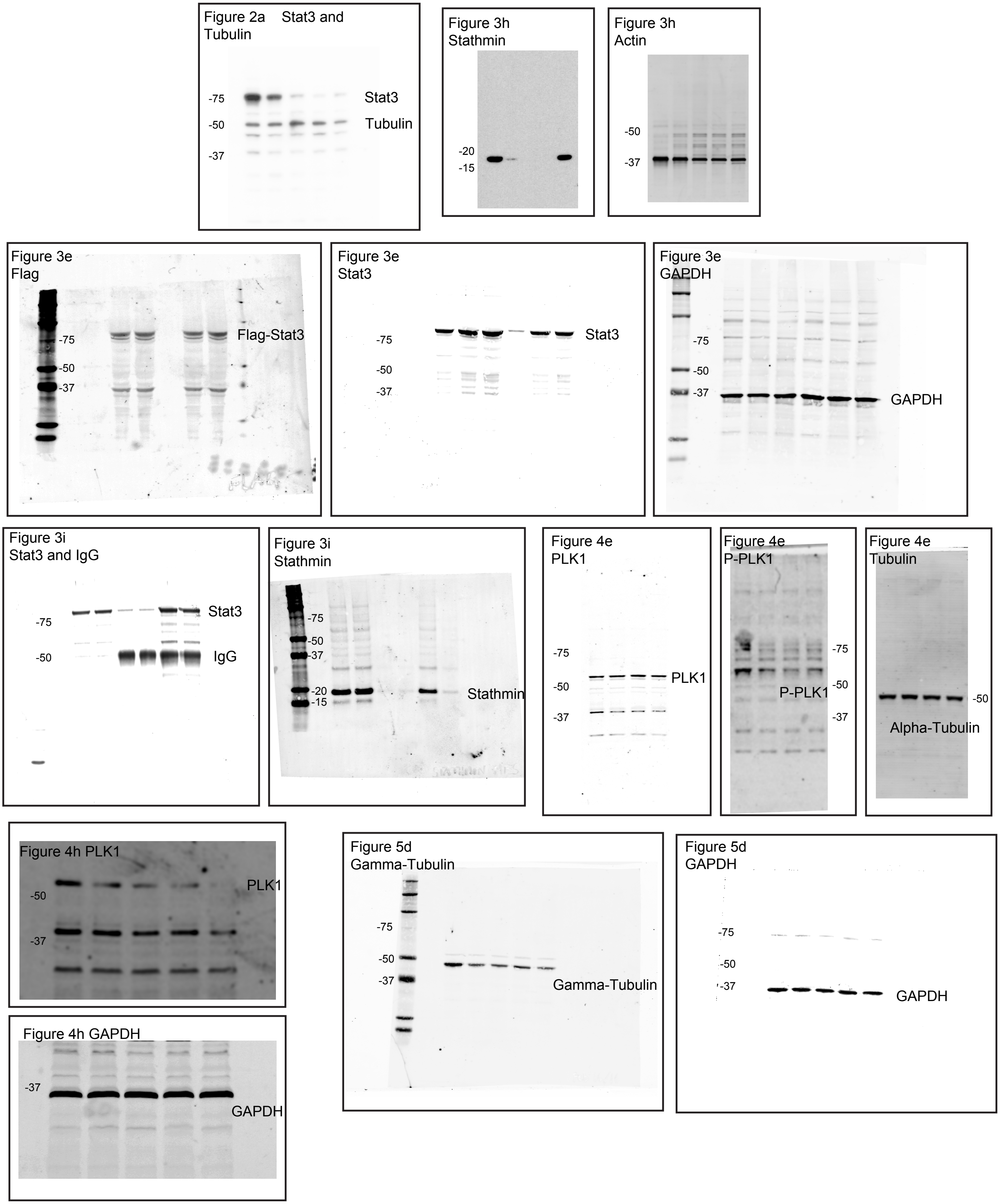

Supplementary Figure 6 Whole blot scans of Western blots used in this paper. Numbers adjacent to Western blots indicate protein markers and are in kilodalton units.
